# Yield of Surveillance Imaging After Mastectomy With or Without Reconstruction for Patients With Prior Breast Cancer: A Systematic Review and Meta-analysis

**DOI:** 10.1001/jamanetworkopen.2022.44212

**Published:** 2022-12-01

**Authors:** Daniel Smith, Setara Sepehr, Andreas Karakatsanis, Fredrik Strand, Antonis Valachis

**Affiliations:** 1 Clinical Epidemiology and Biostatistics, School of Medical Sciences, Örebro University, Örebro, Sweden; 2 School of Medicine, Faculty of Medicine and Health, Örebro University, Örebro, Sweden; 3 Department of Surgical Sciences, Uppsala University, Uppsala, Sweden; 4 Breast Radiology, Karolinska University Hospital, Solna, Sweden; 5 Department of Oncology-Pathology, Karolinska Institute, Solna, Sweden; 6 Department of Oncology, Faculty of Medicine and Health, Örebro University, Örebro, Sweden

## Abstract

**Importance:**

A discrepancy on current guidelines and clinical practice exists regarding routine imaging surveillance after mastectomy, mainly regarding the lack of adequate evidence for imaging in this setting.

**Objective:**

To investigate the usefulness of imaging surveillance in terms of cancer detection and interval cancer rates after mastectomy with or without reconstruction for patients with prior breast cancer.

**Data Sources:**

A comprehensive literature search was conducted in 3 electronic databases—PubMed, ISI Web of Science, and Scopus—without year restriction. References from relevant reviews and eligible studies were also manually searched.

**Study Selection:**

Eligible studies were defined as those conducting surveillance imaging (mammography, ultrasonography, or magnetic resonance imaging [MRI]) of patients with prior breast cancer after mastectomy with or without reconstruction that presented adequate data to calculate cancer detection rates for each surveillance method.

**Data Extraction and Synthesis:**

Independent data extraction by 2 investigators with consensus on discrepant results was performed. A quality assessment of studies was performed using the QUADAS-2 (Quality Assessment of Diagnostic Accuracy Studies–2) template. The generalized linear mixed model framework with both fixed-effects and random-effects models was used to meta-analyze the proportion of cases across studies including 3 variables: surveillance method, reconstruction after mastectomy, and surveillance measure.

**Main Outcomes and Measures:**

Three outcome measures were calculated for each eligible study and each surveillance imaging method within studies: overall cancer detection (defined as ipsilateral cancer, both palpable and nonpalpable) rate per 1000 examinations, clinically occult (nonpalpable) cancer detection rate per 1000 examinations, and interval cancer rate per 1000 examinations.

**Results:**

In total, 16 studies were eligible for the meta-analysis. The pooled overall cancer detection rates per 1000 examinations were 1.86 (95% CI, 1.05-3.30) for mammography, 2.66 (95% CI, 1.48-4.76) for ultrasonography, and 5.17 (95% CI, 1.49-17.75) for MRI. For mastectomy without reconstruction, the rate of clinically occult (nonpalpable) cancer per 1000 examinations (2.96; 95% CI, 1.38-6.32) and the interval cancer rate per 1000 examinations (3.73; 95% CI, 0.84-3.98) were lower than the overall cancer detection rate (including both palpable and nonpalpable lesions) per 1000 examinations (6.41; 95% CI, 3.09-13.25) across all imaging modalities. The interval cancer rate per 1000 examinations for mastectomy with reconstruction (3.73; 95% CI, 0.41-2.73) was comparable to the pooled cancer detection rate per 1000 examinations (4.73; 95% CI, 2.32-9.63) across all imaging modalities. In all clinical scenarios and imaging modalities, lower rates of clinically occult cancer compared with cancer detection rates were observed.

**Conclusions and Relevance:**

Lower detection rates of clinically occult—compared with overall—cancer across all 3 imaging modalities challenge the use of imaging surveillance after mastectomy, with or without reconstruction. Findings suggest that imaging surveillance in this context is unnecessary in clinical practice, at least until further studies demonstrate otherwise. Future studies should consider using the clinically occult cancer detection rate as a more clinically relevant measure in this setting.

## Key Points

**Question:**

Is surveillance imaging necessary for patients with prior breast cancer who have undergone mastectomy?

**Findings:**

In this systematic review and meta-analysis of 16 relevant studies, the pooled overall cancer detection rates were lower than the Breast Imaging Reporting and Data System benchmarks for all 3 imaging modalities (mammography, ultrasonography, and magnetic resonance imaging) both for patients with reconstruction after mastectomy and for patients without reconstruction. In all clinical scenarios and imaging modalities, lower rates of clinically occult (nonpalpable) cancer compared with cancer detection rates were observed.

**Meaning:**

This systematic review and meta-analysis found that the lower detection rates of clinically occult—compared with overall—cancer across all 3 imaging modalities suggest not using imaging surveillance after mastectomy, with or without reconstruction.

## Teaser

This meta-analysis investigates the usefulness of imaging surveillance in terms of cancer detection and interval cancer rates after mastectomy with or without reconstruction for patients with prior breast cancer.

## Introduction

For patients with early breast cancer, 2 general surgical approaches are available as local treatment: breast-conserving surgery (BCS) and mastectomy, the latter of which may be followed with immediate, delayed, or no reconstruction. Although BCS is the most frequently used treatment, many patients undergo mastectomy.^1,2^

Conflicting results exist about the trend of mastectomy rates over time, with population-based studies showing increased,^2^ decreased,^3,4^ or even unchanged^5^ trends. Such discrepancies might reflect the need to consider several factors in the decision-making process for surgery, such as clinicopathologic characteristics, physician- and patient-related factors, and personal preferences.^6^ Irrespective of the temporal trend in mastectomy rates, implant- or tissue-based reconstruction rates among patients with breast cancer treated with mastectomy have steadily increased over time.^2,7,8^

The follow-up surveillance strategy for patients with breast cancer with curative intention aims to detect locoregional recurrences or systemic relapses as well as contralateral breast cancer at an early stage.^9^ Although intensive follow-up strategies, such as laboratory testing and whole-body imaging at regular intervals, have not been found to improve patients’ prognosis or quality of life,^10^ physical examination at regular intervals and annual mammography, with ultrasonography and breast magnetic resonance imaging (MRI) when needed, after BCS are recognized as cost-effective surveillance approaches and are recommended in current international guidelines.^11,12,13^

However, the imaging surveillance strategy for patients with prior breast cancer treated with mastectomy with or without reconstruction is more controversial. The National Comprehensive Cancer Network recommends against routine imaging for patients with breast cancer who have undergone mastectomy with or without reconstruction.^13^ However, the American College of Radiology categorized the use of mammography or digital breast tomosynthesis as potentially appropriate for patients with breast cancer treated with mastectomy and tissue-based reconstruction, but not after mastectomy with implant-based reconstruction or without reconstruction.^14^ These discrepancies in current guidelines reflect the lack of adequate evidence for or against surveillance imaging after mastectomy and might explain why breast imaging seems to be a common practice in contemporary clinical settings.^15,16,17^ Accordingly, there is a need to assess the clinical value of breast imaging in this setting by identifying the detection rate of clinically occult, nonpalpable recurrences that would have been missed through a physical examination.

The purpose of this systematic review and meta-analysis was to examine the yield of surveillance imaging for patients with breast cancer who have undergone mastectomy with or without reconstruction in terms of overall cancer detection rate, clinically occult cancer detection rate, and interval cancer rate.

## Methods

### Search Strategy, Data Extraction, and Quality Assessment

Two investigators (S.S. and A.V.) independently performed the literature search, data extraction, and quality assessment using predefined criteria (eMethods in the Supplement). Consensus was reached between the investigators in case of discrepancies. The Meta-analysis of Observational Studies in Epidemiology (MOOSE) reporting guideline checklist has been used for reporting this study.^18^

### Study Selection Process

The primary objective was structured using the PICO (Population, Intervention, Comparison, Outcome) model: population: patients with previous breast cancer who underwent mastectomy with or without reconstruction (implant or autologous) and *BRCA* (*BRCA1*: OMIM 113705; *BRCA2*: OMIM 600185) variant carriers who have had mastectomy with or without reconstruction (implant or autologous); intervention: imaging diagnostic methods, including mammography, ultrasonography, tomosynthesis, or MRI as surveillance; comparison: patients who were not followed up with imaging surveillance; and outcome: rate of overall cancer, clinically occult cancer, and interval cancer.

We excluded studies with a nonrelevant population (a population other than patients with prior breast cancer after mastectomy or *BRCA* variant carriers after mastectomy), a nonrelevant intervention (surveillance methods other than mammography, ultrasonography, tomosynthesis, and MRI), a nonrelevant outcome (studies that did not present their results on detection rates for each surveillance imaging separately), or studies published in languages other than English.

### Outcomes and Definitions

To make our results comparable, we calculated the cancer detection rates per 1000 examinations. The primary outcome was the pooled cancer detection rate, defined as ipsilateral breast cancer detected with any method and at any period during follow-up. The secondary outcome was the pooled interval cancer rate, defined as ipsilateral cancer detected after a normal imaging procedure but before any subsequent imaging.

An additional analysis of the primary outcome was performed by excluding lesions detected with imaging methods that were clearly described as palpable even without imaging. This outcome was defined as nonpalpable ipsilateral breast cancer detected only with imaging method.

### Data Synthesis

We used the generalized linear mixed model framework to meta-analyze the proportion of cases across studies. The total number of examinations in each study represented the binomial denominator. Fixed categorical effects were modeled as dummy variables and included surveillance method (mammography, ultrasonography, and breast MRI; tomosynthesis was excluded because of insufficient data); reconstruction after mastectomy (no or yes); and surveillance measure (any cancer, clinically occult cancer, and interval cancer). We included the study identifier as a random-effects term to account for the use of multiple observations from the same study. We prespecified the significance threshold for 2-tailed hypothesis tests at α = .05 and accordingly present 95% CIs. Pooled estimates and 95% CIs from the model were presented as the number of cases per 1000 examinations.

## Results

### Study Selection

**Figure 1.**
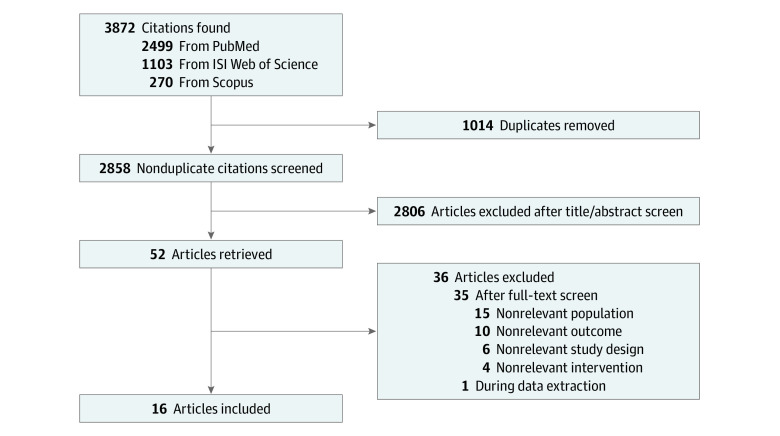
Flowchart Diagram of Study Selection Process

The literature search yielded 2858 articles, of which 52 were selected as potentially eligible after reading the titles and abstracts. From these 52 potentially eligible articles, 16 studies^16,17,19,20,21,22,23,24,25,26,27,28,29,30,31,32^ were considered eligible and included in the analyses. A flowchart diagram of study selection process is illustrated in Figure 1.

### Study Characteristics and Quality Assessment

The characteristics of the eligible studies are presented in the Table. Of 16 studies included, 15 were retrospective cohort studies,^16,17,19,20,21,22,23,24,25,26,27,28,29,30,31^ and 1 was a prospective cohort study.^32^ Six studies used mammography as the surveillance imaging modality,^16,23,28,29,30,32^ 5 used ultrasonography,^17,21,24,25,26^ 3 used MRI,^19,20,27^ and 2 used mammography and ultrasonography at the same time.^22,31^ The time interval for surveillance varied between 6 and 12 months among the eligible studies; however, 5 studies did not report the time interval.^20,27,28,30,31^

**Table.** Characteristics of Eligible Studies

Source	Inclusion period	Study type	Clinical situation	Clinical examination schedule	Surveillance method	Surveillance time interval
Chapman et al,^19^ 2020 (US)	2010-2016	Retrospective	Mastectomy with or without reconstruction	Annually	MRI	12 mo
Golan et al,^20^ 2019 (Israel)	2010-2018	Retrospective	Mastectomy with reconstruction	NR	MRI	NR
Noroozian et al,^16^ 2018 (US)	2000-2015	Retrospective	Mastectomy with reconstruction	Annually	Mammography	12 mo
Liu et al,^21^ 2017 (China)	2006-2008	Retrospective	Mastectomy without reconstruction	NR	Ultrasonography	6 mo
Radhika et al,^22^ 2016 (Malaysia)	NR	Retrospective	Mastectomy without reconstruction	NR	Mammography; ultrasonography	12 mo
Freyvogel et al,^23^ 2014 (US)	2000-2009	Retrospective	Mastectomy with reconstruction	Annually	Mammography	12 mo
Suh et al,^24^ 2013 (South Korea)	2000-2002	Retrospective	Mastectomy without reconstruction	Every 6 mo for 2-3 y and then annually	Ultrasonography	6 mo
Lee et al,^25^ 2013 (South Korea)	2005-2008	Retrospective	Mastectomy without reconstruction	Every 6 mo for 5 y and then annually	Ultrasonography	6-12 mo
Gweon et al,^26^ 2012 (South Korea)	2007-2010	Retrospective	Mastectomy without reconstruction	Every 6 mo for 2-3 y and then annually	Ultrasonography	6 mo
Vanderwalde et al,^27^ 2011 (US)	2003-2009	Retrospective	Mastectomy with reconstruction	NR	MRI	NR
Kim et al,^17^ 2010 (South Korea)	2004-2005	Retrospective	Mastectomy without reconstruction	Annually	Ultrasonography	12 mo
Lee et al,^28^ 2008 (US)	1999-2005	Retrospective	Mastectomy with reconstruction	NR	Mammography	NR
Helvie et al,^29^ 2002 (US)	1997-1999	Retrospective	Mastectomy with reconstruction	NR	Mammography	12 mo
Fajardo et al,^30^ 1993 (US)	1985-1992	Retrospective	Mastectomy with or without reconstruction	In conjunction with mammography	Mammography	NR
Rissanen et al,^31^ 1993 (Finland)	1989-1991	Retrospective	Mastectomy without reconstruction	NR	Mammography; ultrasonography	NR
Stevens et al,^32^ 1969 (US)	NR	Prospective	Mastectomy without reconstruction	Annually	Mammography	12 mo

Abbreviations: MRI, magnetic resonance imaging; NR, not reported.

There were 6 studies that included patients who had undergone mastectomy with reconstruction,^16,20,23,27,28,29^ 8 that included patients with mastectomy without reconstruction,^17,21,22,24,25,26,31,32^ and 2 that included patients with mastectomy with or without reconstruction.^19,30^

No study included only *BRCA* variant carriers, whereas 5 studies included both patients with prior breast cancer and *BRCA* variant carriers after bilateral mastectomy.^16,20,23,27,29^ As a result, no separate data on the potential association of imaging modalities with *BRCA* variant carriers after mastectomy could be pooled, and our study results are restricted to non–*BRCA* variant carriers with prior breast cancer treated with mastectomy.

The questions of the Quality Assessment of Diagnostic Accuracy Studies–2 (QUADAS-2) template were assessed in all 16 eligible studies. eTable 1 in the Supplement shows the quality assessment for each study.

### Cancer Detection Rates per Imaging Modality

The total number of patients and examinations per eligible study as well as the numbers of cancer detections per imaging modality are presented in eTable 2 in the Supplement. The total numbers of patients included in the eligible studies ranged between 48 and 874, and the numbers of examinations ranged between 58 and 5117.

**Figure 2.**
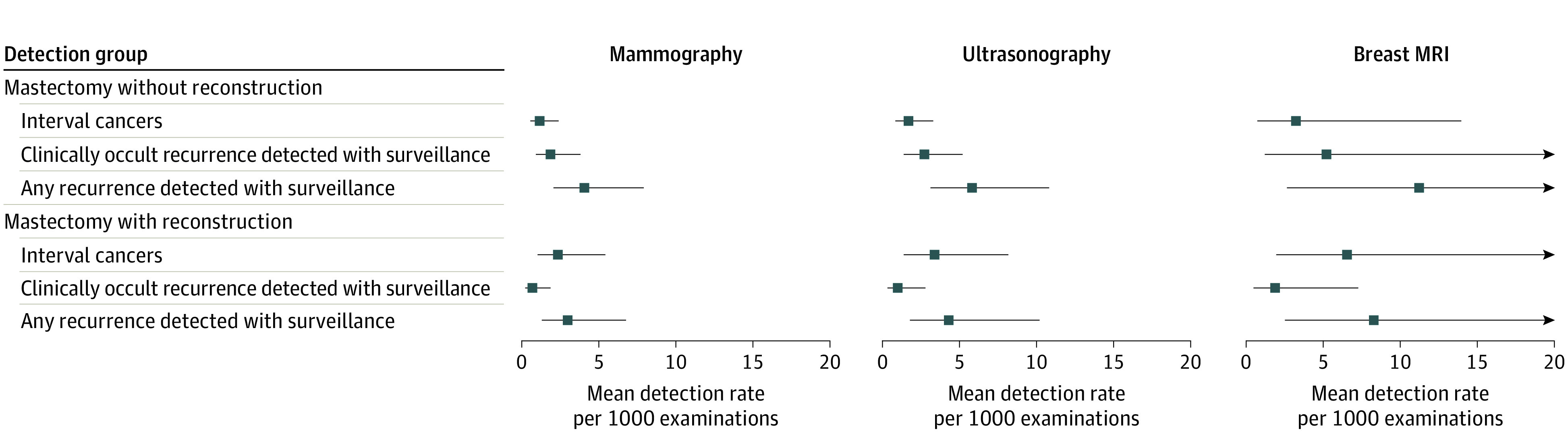
Pooled Cancer Detection Rates per Surveillance Method MRI indicates magnetic resonance imaging.

The pooled overall cancer detection rates per 1000 examinations per imaging modality used were 1.86 (95% CI, 1.05-3.30) for mammography, 2.66 (95% CI, 1.48-4.76) for ultrasonography, and 5.17 (95% CI, 1.49-17.75) for MRI (Figure 2).

### Cancer Detection Rates After Mastectomy With or Without Reconstruction

**Figure 3.**
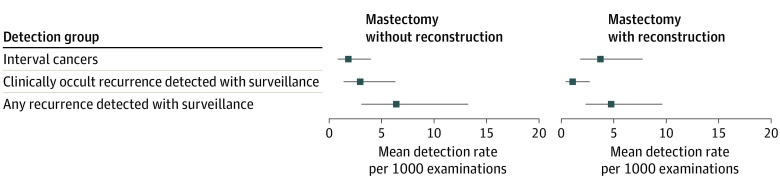
Pooled Cancer Detection Rates, Averaged Over Imaging Modality, Among Patients After Mastectomy Without or With Reconstruction

Figure 3 illustrates the pooled detection rates, averaged over imaging modality, for patients after mastectomy without reconstruction and for those with reconstruction. For mastectomy without reconstruction, the pooled overall cancer detection rate per 1000 examinations was 6.41 (95% CI, 3.09-13.25), whereas the pooled rate of interval cancer per 1000 examinations was 3.73 (95% CI, 0.84-3.98), and the rate of clinically occult (nonpalpable) cancer was 2.96 (95% CI, 1.38-6.32) per 1000 examinations.

For mastectomy with reconstruction, the pooled overall cancer detection rate per 1000 examinations (4.73; 95% CI, 2.32-9.63) was lower than that among patients who underwent mastectomy without reconstruction (Figure 3). The pooled interval cancer rate per 1000 examinations (3.73; 95% CI, 0.41-2.73) was comparable to the pooled cancer detection rate observed among patients who underwent mastectomy with reconstruction. The rate of clinically occult (nonpalpable) cancer per 1000 examinations was 1.06 (95% CI, 0.41-2.73).

### Additional Analyses by Excluding Palpable Lesions at the Time of Imaging

When palpable lesions at the time of imaging were excluded, the pooled clinically occult (nonpalpable) cancer rate per 1000 examinations was 2.96 (95% CI, 1.38-6.32) among patients who underwent mastectomy without reconstruction and 1.06 (95% CI, 0.41-2.73) among patients who underwent mastectomy with reconstruction.

**Figure 4.**
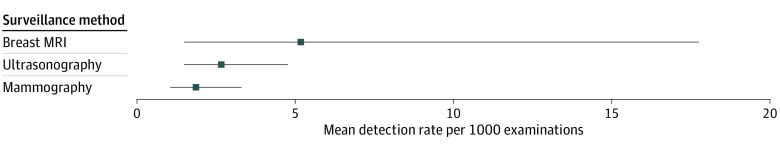
Pooled Cancer Detection Rates in Subgroups Based on Imaging Modality Performed and the Presence of Reconstruction or Not After Mastectomy MRI indicates magnetic resonance imaging.

A subgroup analysis of the pooled detection rates based on reconstruction and imaging modality showed a similar trend on larger differences in clinically occult and interval cancer rates in association with cancer detection rates across all imaging modalities among the patients who underwent mastectomy without reconstruction, whereas interval cancer rates among the patients who underwent mastectomy with reconstruction were comparable to the pooled cancer detection rate across all imaging modalities (Figure 4). For all clinical scenarios and imaging modalities, lower rates of clinically occult cancer compared with cancer detection rates were observed.

### Comparisons Among Surveillance Methods and Outcomes

Prespecified contrasts for select combinations of surveillance method, reconstruction, and outcomes were presented as odds ratios (ORs). For mastectomy without reconstruction, the OR for occult cancer was 0.46 (95% CI, 0.30-0.70; *P* < .001) times the overall cancer detection rate, and the OR for interval cancer was 0.28 (95% CI, 0.18-0.46; *P* < .001) times the overall cancer detection rate. However, the corresponding ORs were not statistically significant for mastectomy with reconstruction; the OR for occult cancer was 0.36 (95% CI, 0.08-1.67; *P* = .08) times the overall cancer detection rate, and the OR for interval cancer was 2.04 (95% CI, 0.53-7.87; *P* = .17) times the overall cancer detection rate. Pooled over the different types of reconstruction, the mean detection rate for ultrasonography was 1.43 (95% CI, 0.88-2.33; *P* = .06) times that of mammography, and the mean detection rate for MRI was 2.79 (95% CI, 0.44-17.84; *P* = .15) times that of mammography, although these rates were not statistically significant.

## Discussion

Although routine imaging surveillance after mastectomy is generally not recommended in international guidelines,^11,12,13^ this approach is still relatively common in daily clinical practice,^15,16,17^ mainly because of the lack of adequate evidence on the potential role of imaging surveillance after mastectomy. Our systematic review and meta-analysis aimed to gather the current evidence in this regard to aid future reviews of these international guidelines

The evidence has been summarized in 2 clinical scenarios: patients who underwent mastectomy without reconstruction and those who underwent mastectomy with reconstruction. We used a clinically relevant definition for the clinically occult cancer detection rate per 1000 examinations (ie, only nonpalpable lesions detected by imaging modalities) that better reflects the real clinical association of imaging surveillance after mastectomy with patients’ prognosis. In fact, a palpable lesion is easier to detect by the patient or the clinician, and any imaging surveillance will probably not affect the lead time and, consequently, the prognosis.^33^ However, a nonpalpable lesion that will be detected by imaging surveillance before it becomes palpable might shorten the lead time and have a potential effect on prognosis.

According to the current evidence, the type of imaging modality does not seem to be associated with cancer detection rates in any of the clinical scenarios examined. Despite the higher mean cancer detection rate with MRI, we were unable to reject the null hypothesis that differences between imaging modalities were due to chance variation. However, in such circumstances of nonsignificance, this does not imply absence of a true effect, so caution is warranted.^34^ A potential difference in the underlying cancer risk among the different patient cohorts that were followed up by different imaging modalities cannot be ruled out as a source of selection bias either. A recent meta-analysis investigating the performance of breast MRI as a surveillance method for patients with a personal history of breast cancer concluded that the evidence is insufficient to recommend for or against the use of MRI in this setting.^35^ That meta-analysis included mostly patients treated with BCS and analyzed the detection rates using the sum of ipsilateral and contralateral cancers, resulting in a higher pooled cancer detection rate compared with our results of 5.17 per 1000 examinations by using breast MRI.

The comparable pooled cancer detection rates between mammography and ultrasonography in our analyses, which argue against the clinical value of ultrasonography in this setting, support the results of current studies showing that the addition of ultrasonography to mammography as a surveillance strategy seems to be associated with higher biopsy rates and costs,^36^ without influencing the sensitivity or the interval cancer rates.^37^

The pooled cancer detection rates among all imaging modalities in our analyses were lower than the current Breast Imaging Reporting and Data System (BI-RADS) benchmarks of 4.7 per 1000 examinations for mammography in the general population, 3.7 per 1000 examinations for ultrasonography in the general population, and 20 per 1000 examinations for breast MRI among women with a hereditary predisposition for breast cancer,^38^ further supporting the notion that the performance of imaging modalities in this setting does not meet clinical performance expectations.

In the clinical scenario of surveillance after mastectomy without reconstruction, the rates of clinically occult, nonpalpable cancer were considerably lower than the cancer detection rates across all imaging modalities. This observation is relevant in clinical practice because it is the nonpalpable lesions detected only with imaging that should be considered when the yield of imaging surveillance is investigated, because palpable lesions will be clinically detected without the need of imaging. Similar trends were observed in the clinical scenario of surveillance after mastectomy with reconstruction. As a result, the clinical value of imaging surveillance after mastectomy is further restricted to the low detection rates of clinically occult, nonpalpable cancer rather than the somewhat higher, but still low in terms of BI-RADS benchmarks,^38^ cancer detection rates.

Considering interval cancer rates, in the clinical scenario of mastectomy with reconstruction, the interval cancer rate was similar to the overall cancer detection rate, whereas this was not the case among patients who underwent mastectomy without reconstruction, where interval cancer rates were lower than overall cancer detection rates. This difference in trends can be explained by the higher cancer detection rates among patients who underwent mastectomy without reconstruction in general rather than by a true difference in the interval cancer rates between the 2 clinical scenarios. In fact, the numerical difference in the cancer detection rates between the 2 clinical scenarios, with higher rates among patients who underwent mastectomy without reconstruction, may reflect the heterogeneity among the eligible studies regarding the number of patients and examinations, the time interval for surveillance, and patients’ baseline characteristics (age, history of breast cancer, and *BRCA* variant carriers) that can introduce a selection bias in the baseline cancer risk of a patient cohort.

The goal of any surveillance strategy is to improve the prognosis of the patients. Although it was beyond the scope of this study to investigate this aspect, we were able to assess the current evidence on the potential role of imaging surveillance regarding prognosis in this setting through our systematic review. Only 2 studies with a limited number of patients presented results on the prognosis of patients with surveillance compared with no surveillance, and the results were contradictory.^15,17^ Shammas et al^15^ could not find any difference in overall survival between patients followed up by any imaging modality after mastectomy and reconstruction and patients without imaging as a follow-up strategy. On the contrary, Kim et al^17^ found a statistically significant difference in overall survival among patients with a diagnosis of ultrasonography-detected asymptomatic recurrence after mastectomy compared with those with a diagnosis of symptomatic recurrence. Considering the very low certainty of evidence as reflected by the limited and contradictory evidence as well as the high risk of bias of the available studies, the question regarding the potential association of imaging surveillance after mastectomy with patients’ prognosis and survival remains unanswered.

### Limitations

This study has several limitations. First, the eligible studies were heterogeneous regarding several important aspects, such as the imaging modality used, surveillance intervals, and patient cohort, thus making comparisons among studies challenging. In an effort to overcome this issue, we expressed and analyzed the results of each study using the same definitions that enable more appropriate comparisons. Second, the quality of most of the eligible studies was relatively low, as is shown using the QUADAS-2 tool, which has influenced the quality of current evidence as well. Third, the inclusion period of eligible study cohorts investigating mammography or ultrasonography as imaging modalities was extremely wide (several decades), thus increasing the risk that the results of individual studies might be influenced by the improvement of imaging modalities through the years. Fourth, we were unable to investigate the potential role of breast tomosynthesis as an imaging modality in this setting because of a lack of evidence. However, studies based mainly on patients treated with BCS have failed to reveal any substantial benefit associated with breast tomosynthesis vs mammography as an imaging surveillance method for patients with prior breast cancer,^39,40^ and there is no rationale why the results would be different for patients with prior breast cancer treated with mastectomy. Fifth, other potentially relevant clinical scenarios, such as *BRCA* variant carriers after bilateral mastectomy or patients with implant-based vs autologous transplant, were not able to be separately summarized and discussed because of the lack of evidence within the eligible studies. Most of the studies included patients with prior breast cancer, whereas only a few included a minority of *BRCA* variant carriers in their cohort. As a result, the summarized evidence on *BRCA* variant carriers should be interpreted with caution. Sixth, the lack of adequate evidence on the association of imaging surveillance with patients’ prognosis precludes any firm conclusion. However, the pooled results regarding cancer detection rates of the present meta-analysis can be used as a proxy for the expected clinical value of imaging surveillance in this setting.

## Conclusions

The current evidence on the potential role of imaging surveillance after mastectomy is limited and challenging to interpret. The lower detection rates of clinically occult, nonpalpable cancer compared with the detection rates of overall cancer across all 3 imaging modalities challenge the use of imaging surveillance after mastectomy with or without reconstruction in clinical practice until more studies are available. Future studies should consider using the detection rate of clinically occult, nonpalpable cancer as a more clinically relevant measure in this setting. Future studies should also focus on specific clinical scenarios for which evidence is lacking and investigate the association of imaging surveillance with prognosis as well as with the cost-effectiveness of surveillance strategies.

## Notes

Supplement.
eMethods.

eReferences.
**eTable 1.** Quality Assessment of Eligible Studies Using QUADAS-2 Tool **eTable 2.** Number of Patients, Examinations, and Cancers Detected With Different Imaging Modalities in Eligible Studies
