# Supplementary material for: Yield of Surveillance Imaging After Mastectomy With or Without Reconstruction for Patients With Prior Breast Cancer: A Systematic Review and Meta-analysis
Source: JAMA Netw Open. 2022 Dec 1;5(12):e2244212. doi: 10.1001/jamanetworkopen.2022.44212 (PMC9716401; doi:10.1001/jamanetworkopen.2022.44212)
Supplement: Supplement. — eMethods. eReferences. eTable 1. Quality Assessment of Eligible Studies Using QUADAS-2 Tool eTable 2. Number of Patients, Examinations, and Cancers Detected With Different Imaging Modalities in Eligible Studies [file jamanetwopen-e2244212-s001.pdf]

Supplementary Online Content

Smith D, Sepehr S, Karakatsanis A, Strand F, Valachis A. Yield of surveillance imaging after mastectomy with or without reconstruction for patients with prior breast cancer: a systematic review and meta-analysis. *JAMA Netw Open.* 2022;5(12):e2244212. doi:10.1001/jamanetworkopen.2022.44212

eMethods.

eReferences.

eTable 1. Quality Assessment of Eligible Studies Using QUADAS-2 Tool

eTable 2. Number of Patients, Examinations, and Cancers Detected With Different Imaging Modalities in Eligible Studies

This supplementary material has been provided by the authors to give readers additional information about their work.

© 2022 Smith D et al. *JAMA Network Open.*

eMethods.

Search strategy, data extraction and quality assessment

Three electronic databases (PubMed, ISI Web of Science, Scopus) were searched without year restriction. A search algorithm was built according to the PICO principle, where different concepts are combined to create search blocks using combinations of the following keywords: breast cancer, BRCA1, BRCA2, BRCA, mammography ultrasound, MRI, magnetic resonance imaging, tomosynthesis, mastectomy, breast reconstruction, autologous breast reconstruction, implant-based reconstruction, follow-up, surveillance. The searching strategy was supplemented with a manual search of reference lists from relevant reviews as well as from eligible studies.

The following information was obtained from each individual study: first author, year of publication, country, type of study (prospective, retrospective), clinical situation (mastectomy with or without reconstruction due to breast cancer or due to BRCA-mutation), type of reconstruction; surveillance method (mammography, ultrasound, tomosynthesis, MRI), interval of surveillance; number of included patients and number of exams, median age, number of breast cancers overall, number of breast cancers found with surveillance, number of palpable and non-palpable breast cancers, number of interval breast cancers; results on local-recurrence free survival, results on overall survival.

The quality of the eligible studies was evaluated based on the quality assessment template for systematic reviews of primary diagnostic accuracy studies, QUADAS-2 (1). QUADAS-2 consists of four domains: patient selection, index text, reference standard and flow and timing. Each domain is evaluated in terms of bias and the first three are also assessed for applicability.

Statistical modelling

The GLMM one-step approach has recently been shown to outperform conventional two-step methods on transformed proportions, which suffer from several limitations (2). The model assumed a binomial error distribution with a logit link function, and estimation of parameters was performed using maximum likelihood (Laplace approximation). Two competing nested models were pre-specified. Model 1 included all fixed predictors as main effect terms, together with an interaction term between reconstruction after mastectomy and surveillance measure. Model 2 was a reduced form of model 1, with the interaction term omitted i.e. only additive main effects were fitted. Model 1 outperformed model 2 (likelihood ratio test: $\chi2 = 19.01$; $P < 0.0001$) and was therefore retained for inference. Statistical analysis was performed using R version 4.1.2 relying heavily on the packages lme4 (3), emmeans (4) and ggplot2 (5).

© 2022 Smith D et al. *JAMA Network Open.*

eReferences.

1. Whiting PF, Rutjes AW, Westwood ME, Mallett S, Deeks JJ, Reitsma JBet al; QUADAS-2 Group. QUADAS-2: a revised tool for the quality assessment of diagnostic accuracy studies. *Ann Intern Med*. 2011;155(8):529-36.
2. Lin L, Chu H. Meta-analysis of Proportions Using Generalized Linear Mixed Models. *Epidemiology*. 2020;31(5):713-717.
3. Bates D, Maechler M, Bolker B, Walker S. Fitting Linear Mixed-Effects Models Using lme4. *Journal of Statistical Software*. 2015;67(1):1-48.
4. Length R (2020). emmeans: Estimated Marginal Means, aka Least-Squares Means. R package version 1.4.8. Available at: https://CRAN.R-project.org/package=emmeans
5. Wickham H. ggplot2: Elegant Graphics for Data Analysis. Springer-Verlag New York, 2016.

© 2022 Smith D et al. *JAMA Network Open.*

eTable 1. Quality Assessment of Eligible Studies Using QUADAS-2 Tool

Author, Year (ref*)	Risk of bias				Applicability concerns		
	Patient selection	Index test	Reference standard	Flow and timing	Patient selection	Index test	Reference standard
Chapman, 2020 (19)	Low	Low	Low	Low	Low	Low	Low
Golan, 2019 (20)	Low	Low	Unclear	Low	Low	Low	Unclear
Noroozian, 2018 (16)	Low	Low	Unclear	Low	Low	Low	Unclear
Liu, 2017 (21)	Low	Unclear	Unclear	Unclear	High	Low	Unclear
Radhika, 2016 (22)	High	Unclear	Unclear	High	Low	Unclear	Unclear
Freyvogel, 2014 (23)	Low	Low	Low	High	Low	Low	Low
Suh, 2013 (24)	Low	Low	Low	Low	Low	Low	Low
Lee, 2013 (25)	Low	Low	Low	Low	Low	Low	Low
Gweon, 2012 (26)	High	Low	Low	Unclear	High	Low	Low
Vanderwalde, 2011 (27)	High	Low	Unclear	Unclear	High	Low	Unclear
Kim, 2010 (17)	Low	Low	Low	Low	Low	Low	Low
Lee, 2008 (28)	Unclear	Low	Low	Unclear	Unclear	Low	Low
Helvie, 2002 (29)	Low	Low	Low	Low	Low	Low	Low
Fajardo, 1993 (30)	Low	Low	Unclear	Unclear	Low	Low	Unclear

© 2022 Smith D et al. *JAMA Network Open.*

Rissanen, 1993 (31)	Low	Low	Unclear	Unclear	Unclear	Low	Unclear
Stevens, 1969 (32)	Unclear	Low	Unclear	High	Unclear	Low	Unclear

*Numbering according to the reference list of the manuscript

© 2022 Smith D et al. *JAMA Network Open.*

eTable 2. Number of Patients, Examinations, and Cancers Detected With Different Imaging Modalities in Eligible Studies

Author, Year (ref*)	N pts	N exams	Cancers detected	Mammography-detected	Ultrasound-detected	Breast MRI-detected	Interval cancers	Palpable lesions at the time of imaging detection
Chapman, 2020 (19)	191	402	6	NA	NA	4	2	0
Golan, 2019 (20)	159	415	4	NA	NA	1	3	NR
Noroozian, 2018 (16)	485	4163	13	8	NA	NA	5	3
Liu, 2017 (21)	324	5117	9	NA	9	NA	0	NR
Radhika, 2016 (22)	183	183	2	0	2	NA	0	0
Freyvogel, 2014 (23)	397	2131	2	2	NA	NA	0	2
Suh, 2013 (24)	286	2925	6	NA	6	NA	0	1
Lee, 2013 (25)	468	1180	11	NA	10	NA	1	0
Gweon, 2012 (26)	NR	2681	15	NA	15	NA	0	9
Vanderwalde, 2011 (27)	48	58	2	NA	NA	0	2	0
Kim, 2010 (17)	874	1796	19	NA	15	NA	4	0
Lee, 2008 (28)	264	554	0	0	NA	NA	0	0
Helvie, 2002 (29)	113	214	3	3	NA	NA	0	2
Fajardo, 1993 (30)	827	1616	39	20	NA	NA	19	0
Rissanen, 1993 (31)	833	4165	55	50	17	NA	5	11
Stevens, 1969 (32)	210	670	13	9	NA	NA	4	10

Abbreviations: MRI, magnetic resonance imaging; NA, not applicable; NR, not reported.

*Numbering according to the reference list of the manuscript

© 2022 Smith D et al. *JAMA Network Open.*
